# B Cell-Related Circulating MicroRNAs With the Potential Value of Biomarkers in the Differential Diagnosis, and Distinguishment Between the Disease Activity and Lupus Nephritis for Systemic Lupus Erythematosus

**DOI:** 10.3389/fimmu.2018.01473

**Published:** 2018-06-29

**Authors:** Huidi Zhang, Xixi Huang, Lulu Ye, Gangqiang Guo, Xiao Li, Chaosheng Chen, Li Sun, Baoqing Li, Nan Chen, Xiangyang Xue

**Affiliations:** ^1^Department of Nephrology, Ruijin Hospital, Shanghai Jiaotong University School of Medicine, Shanghai, China; ^2^School of the 2nd Clinical Medical Sciences, Wenzhou Medical University, Wenzhou, Zhejiang, China; ^3^Department of Microbiology and Immunology, Institute of Molecular Virology and Immunology, Institute of Tropical Medicine, Wenzhou Medical University, Wenzhou, Zhejiang, China; ^4^Department of Nephrology of the First Affiliated Hospital, Wenzhou Medical University, Wenzhou, Zhejiang, China; ^5^Department of Rheumatology of the First Affiliated Hospital, Wenzhou Medical University, Wenzhou, Zhejiang, China; ^6^Department of Laboratory Medicine of the Second Affiliated Hospital, Wenzhou Medical University, Wenzhou, Zhejiang, China

**Keywords:** systemic lupus erythematosus, circulating microRNA, diagnosis, biomarker, miR-15b

## Abstract

Our understanding of circulating microRNAs (miRNAs) related to systemic lupus erythematosus (SLE) remains very limited. In this study, we screened SLE-specific miRNAs in plasma from 42 B cell-related miRNAs by using miRNA PCR Array. The selected miRNAs were first confirmed in plasma samples from 50 SLE patients, 16 rheumatoid arthritis (RA) patients, and 20 healthy donors using qRT-PCR. We then investigated the relationship between expressions of the selected miRNAs and SLE clinical indicators. As a result, 14 miRNAs (miR-103, miR-150, miR-20a, miR-223, miR-27a, miR-15b, miR-16, miR-181a, miR-19b, miR-22, miR-23a, miR-25, miR-92a, and miR-93) were significantly decreased in the plasma of SLE patients compared with healthy controls (*P* < 0.05) and could act as the diagnostic signature to distinguish SLE patients from healthy donors. Six miRNAs (miR-92a, miR-27a, miR-19b, miR-23a, miR-223, and miR-16) expressed in plasma were significantly lower in SLE patients than in RA patients (*P* < 0.05), revealing the potentially diagnostic signature to distinguish SLE patients from RA patients. Furthermore, the downregulated expression of miR-19b, miR-25, miR-93, and miR-15b was associated with SLE disease activity (*P* < 0.05) while miR-15b and miR-22 expressions were significantly lower in SLE patients with low estimate glomerular filtration rate (eGFR < 60 ml/min/1.73 m^2^) (*P* < 0.05). The diagnostic potential of miR-15b for SLE disease activity and lupus nephritis (LN) with low eGFR was validated on an independent validation set with 69 SLE patients and a cross-validation set with 80 SLE patients. In summary, the signature of circulating miRNAs will provide novel biomarkers for the diagnosis of SLE and evaluation of disease activity and LN.

## Introduction

Systemic lupus erythematosus (SLE) is a clinically heterogeneous autoimmune disease which affects multiple organs and systems and causes significant morbidity and mortality (1). Recently, several criteria have been developed to diagnose SLE or determine SLE activity at an early phase (2). However, owing to its pathogenesis and the exact etiology that has not been completely elucidated, to discover novel biomarkers for early diagnosis of this disease and predicting the therapeutic outcome is very urgent, which enables clinicians to treat SLE patients with the most optimally biologic therapy as early as possible.

MicroRNAs (miRNAs) are a class of non-coding small RNAs approximately having 19–25 nucleotides. miRNAs play key roles in regulating post-transcriptional gene expression by complementary pairing to their target messenger RNAs (3, 4). Over the last decade, published studies have provided strong evidence for a connection between expression of dysregulated miRNAs and development of several systemic autoimmune diseases, including SLE, which gives us new insights into the pathogenesis of SLE and a new opportunity to find novel diagnostic or therapeutic targets (5–13). In addition, miRNAs were present in serum or plasma in a remarkably stable form, and prevented degradation from endogenous RNase activity (14–17). Thus, cell-free circulating miRNAs will be important biomarkers for SLE diagnosis.

It has been demonstrated that uncontrolled over-activated B cells through their maturation into antibody-producing plasma cells are central to the pathogenesis and development of SLE (9, 18). B cells also regulate T-cell activity and immune response by acting as antigen-presenting cells or *via* the production of co-stimulatory molecules and proinflammatory cytokines. Therefore, B cell-related miRNAs may be an attractive target for SLE diagnosis and sequent evaluation of therapeutic outcome.

We have previously screened 72-miRNAs profiling differently expressed in B cells of SLE patients using Affymetrix miRNA 2.0 array (19). In this study, expression of 42 selected miRNAs in a large set of plasma specimens from patients with SLE and rheumatoid arthritis (RA) as well as from healthy controls (HCs) were analyzed using qRT-PCR aiming to identify the miRNAs that could potentially serve as novel serum-based biomarkers for the diagnosis of SLE and further distinguishment between disease activity and lupus nephritis (LN).

## Materials and Methods

### Participants and Study Design

Fifty SLE patients and 16 RA cases receiving clinical care at Department of Rheumatology, the First Affiliated Hospital of Wenzhou Medical University, were enrolled in this study between March 2013 and September of 2015. The plasma of 30 age- and sex-matched HCs were recruited from the Wenzhou local blood bank diagnosed without arthralgia, heart failure, renal failure, or autoimmune disease and free from other inflammatory conditions. Sixty-nine patients in the independent validation cohort were enrolled from March 2013 to September 2015. Additional 80 SLE patients in cross-validation set were enrolled between March 2013 and September 2015 from the Department of Nephrology, Ruijin Hospital of Shanghai Jiao Tong University School of Medicine. The research protocol had been approved by the Medical Ethics Committee of First Affiliated Hospital of Wenzhou Medical University and Ruijin Hospital of Shanghai Jiao Tong University School of Medicine. All participants were informed and gave their written consent to participate in the study. All SLE/RA patients fulfilled the American College of Rheumatology criteria for SLE/RA (20, 21). Disease activity was assessed by the Systematic Lupus Activity Measure and Systemic Lupus Erythematosus Disease Activity Index (SLEDAI) at the time of plasma collection. The SLE patients with SLEDAI >15 were polled together and set as highly active SLE group, those SLE patients with SLEDAI <4 were polled and set as stable SLE group. Modification of diet in renal disease formula was used to estimate glomerular filtration rate (eGFR). All the patient’s clinical features and treatment drugs were collected: drugs for SLE include steroids, mycophenolate mofetil, cyclophosphamide, azathioprine, MTX, tacrolimus, cyclosporine A, and Leflunomide.

**Figure 1:**
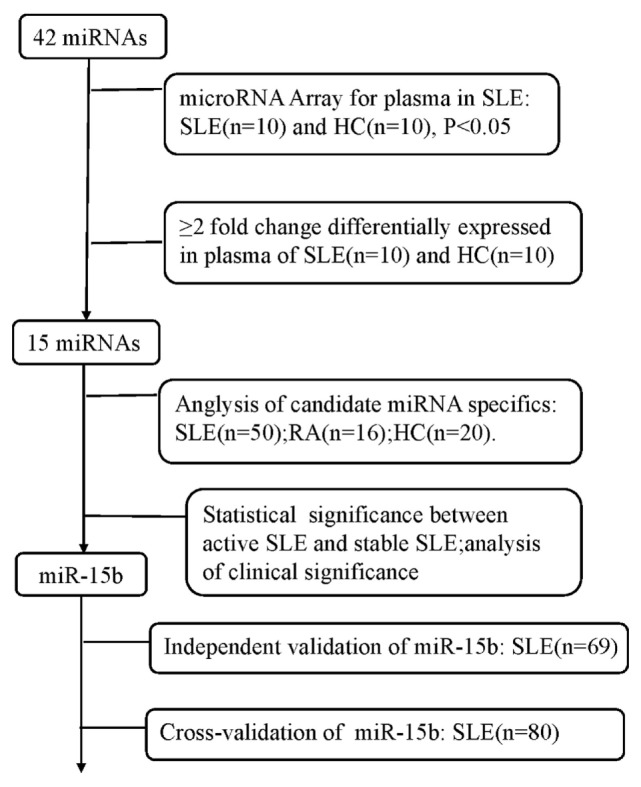
A flowchart of the study design.

The study was designed as a three-phase epidemiological approach to identify the novel circulating miRNAs associated with the disease status of SLE (Figure 1). (1) 10 plasma samples randomly selected from the enrolled SLE patients and 10 plasmas from the age- and sex-matched HCs were used to screen SLE-specific circulating miRNAs from 42 SLE B cell-associated miRNAs using customized miRNAs qPCR array. (2) Different expression of candidate circulating miRNAs was confirmed in the plasma samples comprised of 50 SLE patients, 20 HCs, and 16 patients with RA using qRT-PCR. (3) The identified miRNAs related to the disease activity and LN of SLE were re-validated on two independent SLE cohorts. The Wenzhou validation cohort included 69 SLE patients, and the Shanghai cross-validation cohort consisted of 80 SLE patients. Basic information of all participants was shown in Tables 1 and 2.

**Table 1:** Clinical details of the patients with systemic lupus erythematosus (SLE), rheumatoid arthritis (RA), and healthy controls (HCs).

Clinical characteristic	SLE	RA	HC
Number of patients	50	16	20
Sex, male/female	10/40	3/13	6/14
Age	37.18 ± 15.06	47.62 ± 10.43	
Systemic Lupus Erythematosus Disease Activity Index (SLEDAI), mean ± SD (range)	6.98 ± 5.38 (0–20)		
Erythrocyte sedimentation rate	15 (39)	14 (16)	
The stage of chronic kidney disease			
Early stage (CKD 1~2 stage)	29 (34)		
Late stage (CKD 3~5 stage)	5 (34)		
Autoantibodies and complement			
ANA	40 (40)	6 (11)	
Anti-double-stranded DNA	17 (40)	0 (11)	
Low C3 level	32 (46)	1 (12)	
Low C4 level	26 (41)	2 (12)	
Low *P* level	4 (24)	2 (8)	
C-reactive protein (mg/l)	3.34 ± 6.32	41.01 ± 63.17	
Treatment drugs			
Steroids	50		
Immunosuppressive drugs	28		

*In the phase of candidate microRNA selection, the 10 samples were from 50 samples of SLE patients*.

**Table 2:** Clinical features of systemic lupus erythematosus cases on independent validation set from Wenzhou and cross-validation set from Shanghai.

Clinical characteristic	Independent validation set	Independent validation set
Number of patients	69	80
Sex, male/female	9/60	13/67
Age	32.97 ± 11.71	38.96 ± 14.66
Incipient	14 (69)	30 (80)
Systemic Lupus Erythematosus Disease Activity Index (SLEDAI), mean ± SD (range)	13.30 ± 6.82 (0–30)	10.66 ± 7.97 (0–33)
Erythrocyte sedimentation rate	35 (61)	
The stage of chronic kidney disease		
Early stage (CKD 1~2 stage)	64 (69)	60 (80)
Late stage (CKD 3~5 stage)	5 (69)	20 (80)
Autoantibodies and complement		
ANA	69 (69)	71
Anti-double-stranded DNA	42 (58)	54
Low C3 or C4 level	52 (67)	41 (79)
Low C4 level	50 (63)	53
Low *P* level	7 (37)	2
C-reactive protein (mg/l)	12.69 ± 18.87	23.98 ± 54.74
Treatment drugs		
Steroids	67	77
Immunosuppressive drugs	46	61

*Disease manifestations were defined and scored according to the SLEDAI*.

### Preparation of Plasma Samples and RNA Isolation

Blood samples were collected into EDTA-2K containing tubes. Blood cells were removed by a two-step centrifugation protocol performed at 4°C (3,000 rpm for 10 min, then 12,000 rpm for 10 min) to obtain platelet-poor, cell-free plasma. All samples were stored at −80°C until analyzed. Plasma RNA was isolated using Trizol^®^ LS Reagent (Ambion, US) and Direct-zol™ RNA kit (Cat#2060&2062, Zymo Research, US) according to the manufacturer’s protocol. To normalize possible sample-to-sample variation caused by RNA isolation, 1 nmol (total volume of 1 µl) of synthetic *C. elegans* miRNAs cel-miR-39 (MIMAT0000010, Seq: 5′-UCACCGGGUGUAAAUCAGCUUG-3′, GenePharma, China) was added to each denatured sample. The isolated RNAs were digested by Dnase I cocktail to remove the residual DNA, and then were collected in 25 μl DNase/RNase-free water using Direct-zol™ RNA kit. Concentration of RNA was measured by NanoDrop 2000 (Thermo Fisher scientific, US). Isolated RNA was kept at −80°C or immediately used for reverse transcription.

### Screening of B Cell-Related miRNAs in SLE Plasma Using the Customized qRT-PCR miRNA Array

Two independent experiments were performed to detect the different expressions of B cell-related miRNAs in SLE plasma using the customized miRNAs array (CT Bioscience, Jiangsu, China). To eliminate the difference among different samples as well as possible, five plasma samples from SLE patients or HC were mixed as one pool. The panel of the customized miRNA array contained 42 B cell-related miRNAs selected from our previous study (19). First, aliquot 100 ng of plasma RNA was added the poly A tail and then transcribed into cDNA according to the manufacturer’s protocol. qRT-PCR detection using the customer PCR chip contained 42 selected miRNA panels was performed on an Applied BioSystems 7500 Real-Time PCR system (Life Technologies). Data were automatically analyzed with SDS Relative Quantification Software version 2.2.2 (Life Technologies). U6, let-7d and let-7g were set as internal controls for plasma miRNA (22), and cel-miR-39 was set as the external control for plasma RNA isolation. The miRNAs that the Ct values were greater than 35 or with unqualified dissolution curve were removed from all datasets. Average Ct values of the duplicate analysis of each miRNA were then subtracted from the average Ct value of the cel-miR-39 or the internal control of U6, let-7d and let-7g for that particular sample, yielding the ΔCt values. The relative expression level of each miRNA was measured through the equation 2^−ΔCt^. The experiment was repeated three times.

### Detection of miRNAs Expression

Plasma miRNA expressions of 119 samples from the First Affiliated Hospital of Wenzhou Medical University were detected. First, 100 ng plasma RNA was reversely transcribed using miScript II RT kit (Qiagen, Valencia, German) according to the manufacturer’s protocol. Stem-loop Real-time qPCR was performed using the miScript SYBR Green PCR Kit (Cat#218073, Qiagen, German) according to the manufacturer’s protocol. The expressions of miRNAs in the 80 plama samples from Ruijin Hospital were detected using mirVana qRT-PCR miRNA Detection Kit (Cat#AM1558, Ambion, US). All RT-qPCR reactions were carried out on an Applied BioSystems 7500 Real-Time PCR system (Life Technologies). All reactions ran in triple. The data were analyzed through the comparative threshold cycle (Ct) method. For expression analysis, the experiment was designed to use the cel-miR-39 as the external control; therefore, the relative quantification of plasma miRNAs was calculated using the equation: amount of target miRNA expression = 2^−ΔCt^, ΔCt = Ct_plasma miRNAs_ − Ct_cel-miR-39_. The results were magnified 10,000 times.

### Statistical Analysis

Data were presented as the median and quartile. SPSS 22.0 software was applied for statistical analysis. Statistical significance between groups was determined by Mann–Whitney *U*-test, Student’s *t*-test, chi-square test, Fisher’s exact test, Pearson product-moment correlation coefficient, and logistic regression analysis with Dunnett’s multiple comparison test as appropriate. *P* value less than 0.05 was considered statistically significant. Receiver operating characteristic (ROC) curve analysis, plotting the true positive rate (sensitivity) versus the false positive rate (1 − specificity) at various threshold settings were performed for plasma miRNAs, and the areas under curve (AUC) was calculated with SPSS22.0. The maximum of the sum of true positive rate and false positive rate were calculated, and cutoff value with higher specificity was selected. Expression graphs and ΔCt values were analyzed using GraphPad Prism version 5.04 software.

## Results

### Screening and Verifying SLE-Specific Circulating miRNAs

**Figure 2:**
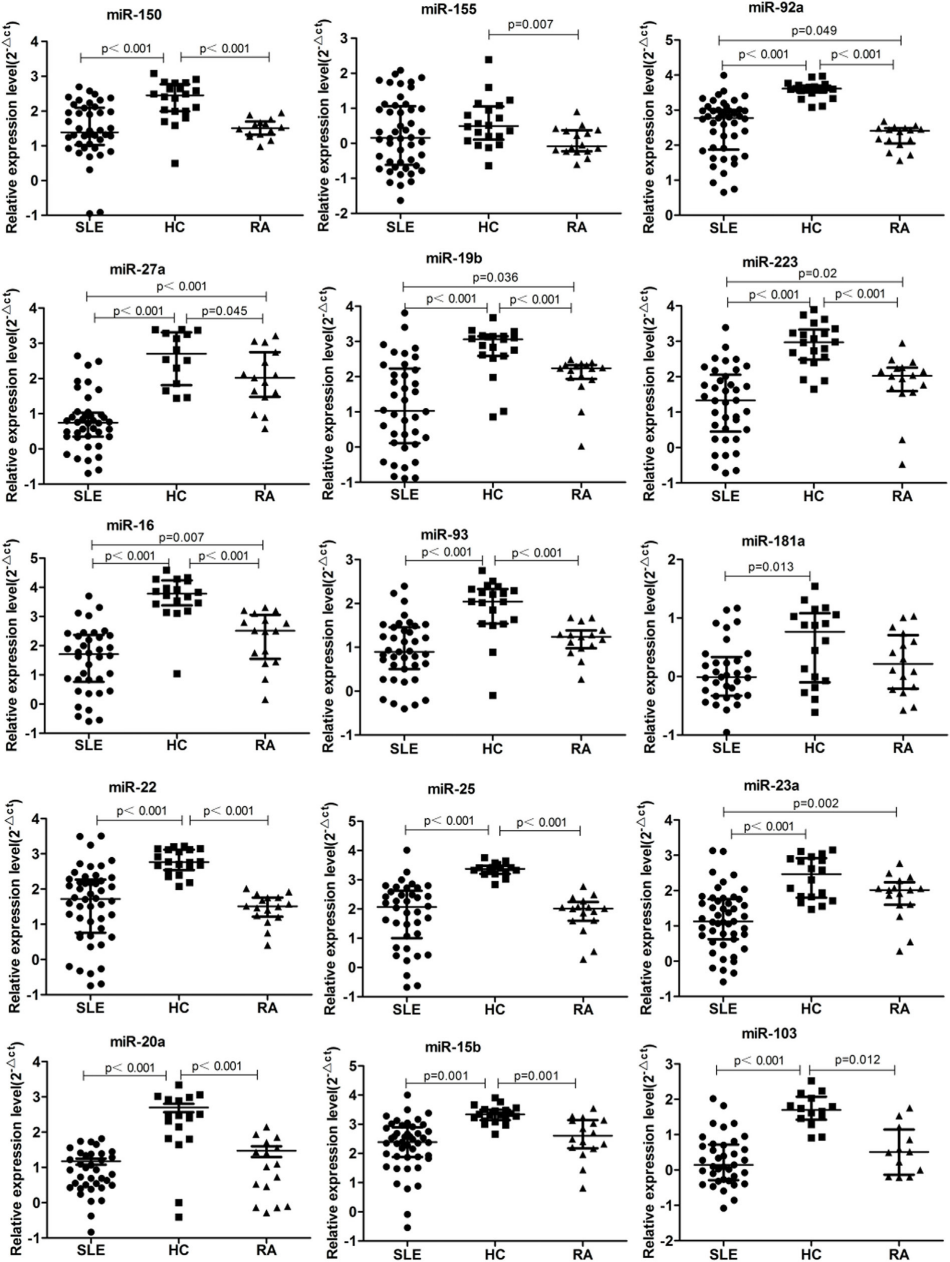
15 microRNA (miRNA) differentially expressed in the plasma of systemic lupus erythematosus (SLE), rheumatoid arthritis (RA) patients, and healthy control (HC). Expressions of the selected miRNAs in the plasma obtained from patients with SLE (*n* = 50), RA (*n* = 16), and HC (*n* = 20) were determined by qRT-PCR. The expression levels of miRNAs were normalized to cel-miR-39.

According to our previous results of miRNAs profiling differentially expressed in B cells of SLE patients and combined with reported literature, 42 B cell-associated miRNAs (Tables S1–S4 in Supplementary Material) were selected to customize an miRNAs panel array for detecting plasma miRNAs associated with SLE. Results from the two set of experiments revealed that expression of 15 miRNAs had twofold change in the plasma between SLE patients and HC. One miRNA, miR-155, was upregulated in the plasma of SLE patients, and the other 14 miRNAs (miR-103, miR-150, miR-20a, miR-223, miR-27a, miR-15b, miR-16, miR-181a, miR-19b, miR-22, miR-23a, miR-25, miR-92a, and miR-93) were downregulated (Table 3). Based on the screening results of SLE-specific circulating miRNAs, we further analyzed the expressions of 15 selected miRNAs in plasma of 50 SLE patients, 16 RA patients, and 20 HCs. As shown in Figure 2, expressions of 14 miRNAs (miR-103, miR-150, miR-20a, miR-223, miR-27a, miR-15b, miR-16, miR-181a, miR-19b, miR-22, miR-23a, miR-25, miR-92a, and miR-93) were significantly downregulated in the plasma of SLE patients compared with those in HCs (*P* < 0.05), consistent with the result of our customized miRNAs panel array screening. The expression of miR-155 showed no significant difference in the plasma between SLE patients and HCs (*P* > 0.05). Furthermore, except for miR-181a, 13 miRNAs were also significantly downregulated in the plasma of RA patients compared with HCs (*P* < 0.05). In addition, six miRNAs (miR-23a, miR-92a, miR-223, miR-27a, miR-16, and miR-19b) in the plasma of SLE patients were significantly lower than those in RA patients (*P* < 0.05).

**Table 3:** Differential expression of B cells-associated microRNAs (miRNAs) in the plasma of systemic lupus erythematosus (SLE) patients and healthy control (HC).

miRNA	Fold change (SLE/HC, 2^**−Δ**ct^) A				miRNA	Fold change (SLE/HC, 2^**−Δ**ct^) B			
	U6	let-7d	let-7g	cel-miR-39		U6	let-7d	let-7g	cel-miR-39
miR-155	5.59	1.98	4.43	3.03	miR-155	12.99	14.92	12.12	14.92
miR-103	0.61	0.21	0.48	0.33	miR-103	0.08	0.1	0.08	0.1
miR-150	0.51	0.18	0.41	0.28	miR-150	0.7	0.81	0.65	0.81
miR-20a	0.63	0.22	0.5	0.34	miR-20a	0.61	0.7	0.57	0.7
miR-223	0.65	0.23	0.51	0.35	miR-223	0.28	0.32	0.26	0.32
miR-27a	0.57	0.2	0.45	0.31	miR-27a	0.4	0.46	0.37	0.46
miR-15b	0.03	0.01	0.03	0.02	miR-15b	0.003	0.004	0.003	0.004
miR-16	0.01	0.01	0.01	0.01	miR-16	0.01	0.01	0.01	0.01
miR-181a	0.09	0.03	0.07	0.04	miR-181a	0.28	0.32	0.26	0.32
miR-19b	0.19	0.07	0.15	0.1	miR-19b	0.16	0.18	0.15	0.18
miR-22	0.01	0	0.01	0.01	miR-22	0.1	0.11	0.09	0.11
miR-23a	0.22	0.07	0.17	0.12	miR-23a	0.1	0.11	0.09	0.11
miR-25	0.16	0.05	0.12	0.08	miR-25	0.46	0.53	0.43	0.53
miR-92a	0.04	0.01	0.03	0.02	miR-92a	0.04	0.05	0.04	0.05
miR-93	0.03	0.01	0.02	0.01	miR-93	0.16	0.18	0.15	0.18

*miRNA relative expression in the two cohorts’ plasma of SLE and HC. Cel-miR-39 is an external reference, U6 is the most common internal reference, and let-7d and let-7g are common internal reference. The fold change in the columns is the ratio of expression in patients with SLE versus controls when using different normalization. All P values were less than 0.05*.

### ROC Curve Analysis of 14 Candidate Plasma miRNAs as SLE Diagnosis

**Figure 3:**
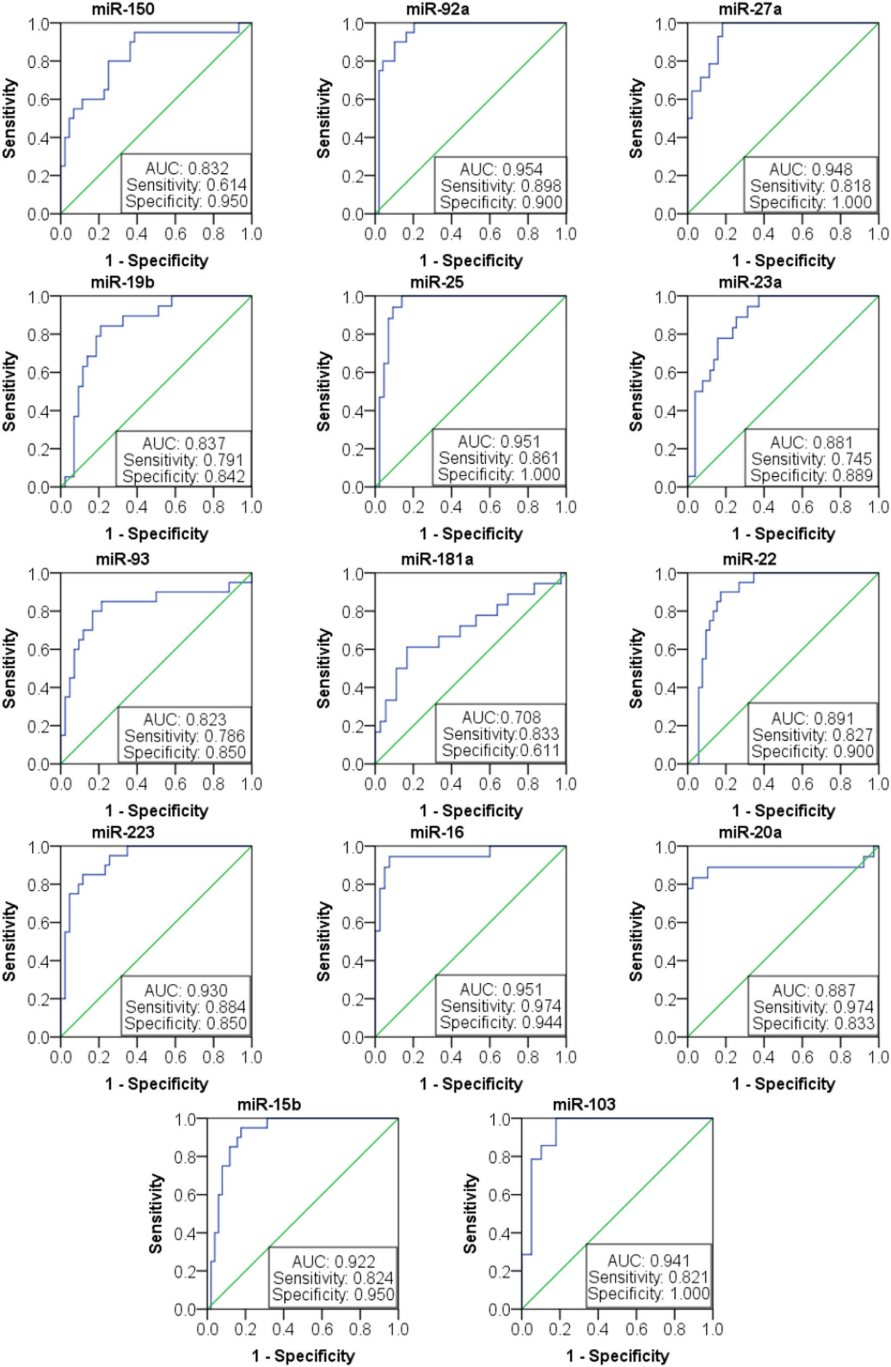
Receiver operating characteristic (ROC) curve of 14 microRNAs (miRNAs) for systemic lupus erythematosus (SLE) diagnosis from healthy control. The areas under curve (AUC) for miRNAs are shown in Figure 3 and Table 4, respectively. The diagnostic sensitivity for SLE difference from healthy people was 0.61~0.97 and the specificity was 0.61~1.

**Figure 4:**
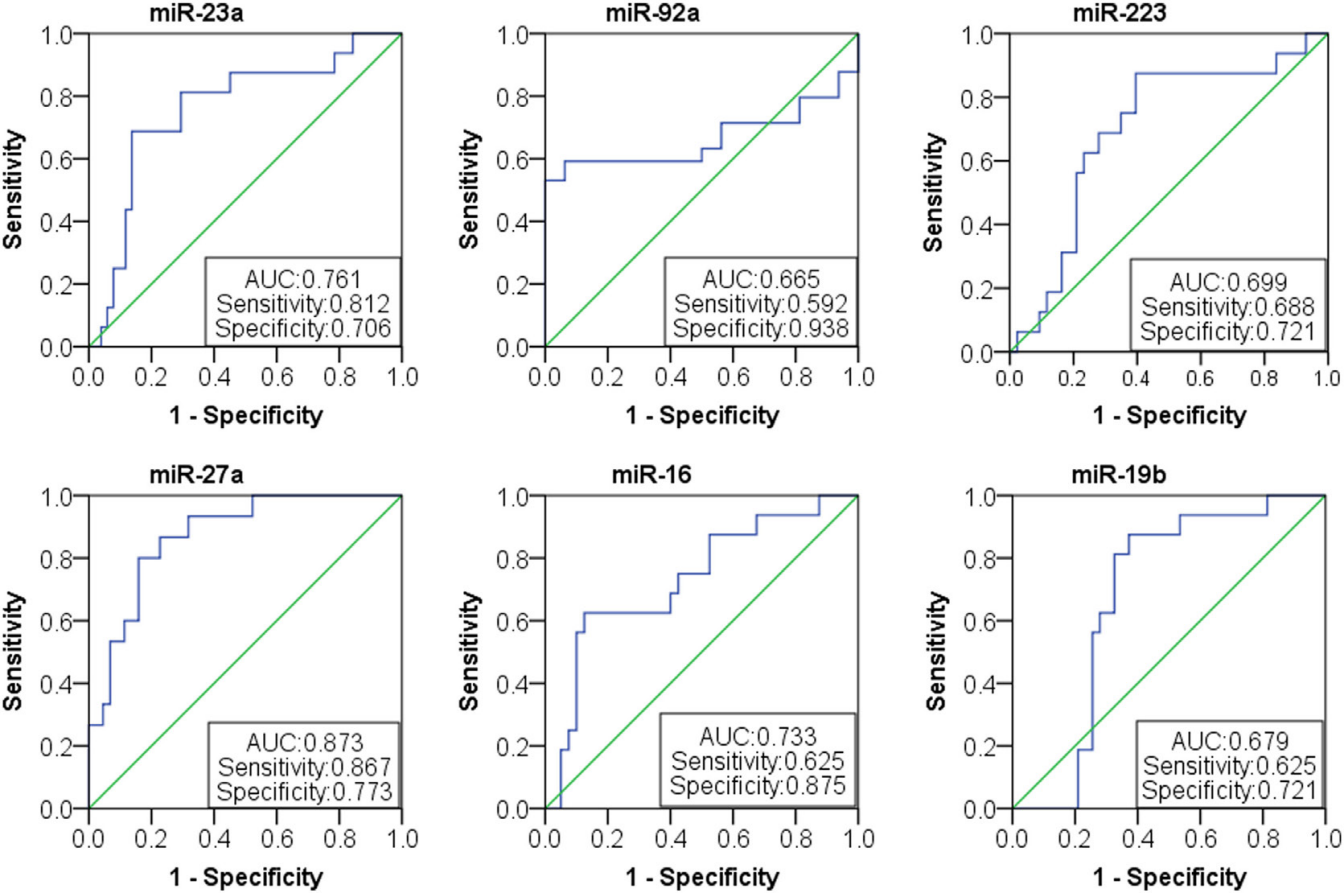
Receiver operating characteristic (ROC) curve of six microRNAs (miRNAs) for systemic lupus erythematosus (SLE) diagnosis from rheumatoid arthritis patients. The areas under curve (AUC) for miRNAs are shown in Figure 4 and Table 5, respectively. The diagnostic sensitivity for SLE difference from healthy people was 0.59~0.87 and the specificity was 0.71~0.94.

Based on the results of SLE-specific circulating miRNA, we investigated whether miRNAs could be used as a new diagnostic marker of SLE using the ROC curve. As shown in Figure 3 and Table 4, the AUC for miR-150, miR-92a, miR-27a, miR-19b, miR-25, miR-23a, miR-93, miR-181a, miR-22, miR-223, miR-16, miR-20a, miR-15b, and miR-103 in predicting SLE from HCs were 0.832, 0.954, 0.948, 0.837, 0.951, 0.881, 0.823, 0.708 0.891, 0.93, 0.951, 0.887, 0.922, and 0.941, respectively. The diagnostic sensitivity for SLE distinguish from HCs was 0.61~0.97 and the specificity was 0.61~1. In addition, the AUCs for miR-92a, miR-27a, miR-19b, miR-223, miR-23a, and miR-16 in predicting SLE from RA were 0.665, 0.873, 0.679, 0.699, 0.761, and 0.733, respectively (Figure 4; Table 5). The diagnostic sensitivity for SLE distinguish from RA patients was 0.59~0.87 and the specificity was 0.71~0.94.

**Table 4:** Receiver operating characteristic (ROC) curve (AUC) for 14 microRNAs (miRNAs) in discriminating systemic lupus erythematosus patients from healthy control.

miRNA	ROC area (AUC)	95% upper bound	95% lower bound	Specificity	Sensitivity
miR-150	0.832	0.946	0.718	0.95	0.614
miR-92a	0.954	1.003	0.905	0.9	0.898
miR-27a	0.948	1	0.896	1	0.818
miR-19b	0.837	0.941	0.733	0.842	0.791
miR-25	0.951	1.006	0.896	1	0.861
miR-23a	0.881	0.961	0.802	0.889	0.745
miR-93	0.823	0.957	0.688	0.85	0.786
miR-181a	0.708	0.869	0.548	0.611	0.833
miR-22	0.891	0.968	0.815	0.9	0.827
miR-223	0.93	0.992	0.869	0.85	0.884
miR-16	0.951	1.02	0.883	0.944	0.974
miR-20a	0.887	1.029	0.746	0.833	0.974
miR-15b	0.922	0.984	0.859	0.95	0.824
miR-103	0.941	1.002	0.881	1	0.821

**Table 5:** Receiver operating characteristic (ROC) curve (AUC) for six microRNAs (miRNAs) in discriminating systemic lupus erythematosus patients from rheumatoid arthritis patients.

miRNA	ROC area (AUC)	95% upper bound	95% lower bound	Specificity	Sensitivity
miR-92a	0.665	0.790	0.539	0.938	0.592
miR-27a	0.873	0.966	0.779	0.773	0.867
miR-19b	0.679	0.818	0.540	0.721	0.625
miR-223	0.699	0.850	0.549	0.721	0.688
miR-23a	0.761	0.900	0.622	0.706	0.812
miR-16	0.733	0.882	0.583	0.875	0.625

### Correlation Between Plasma miRNAs Expression and SLE Clinical Variables

**Figure 5:**
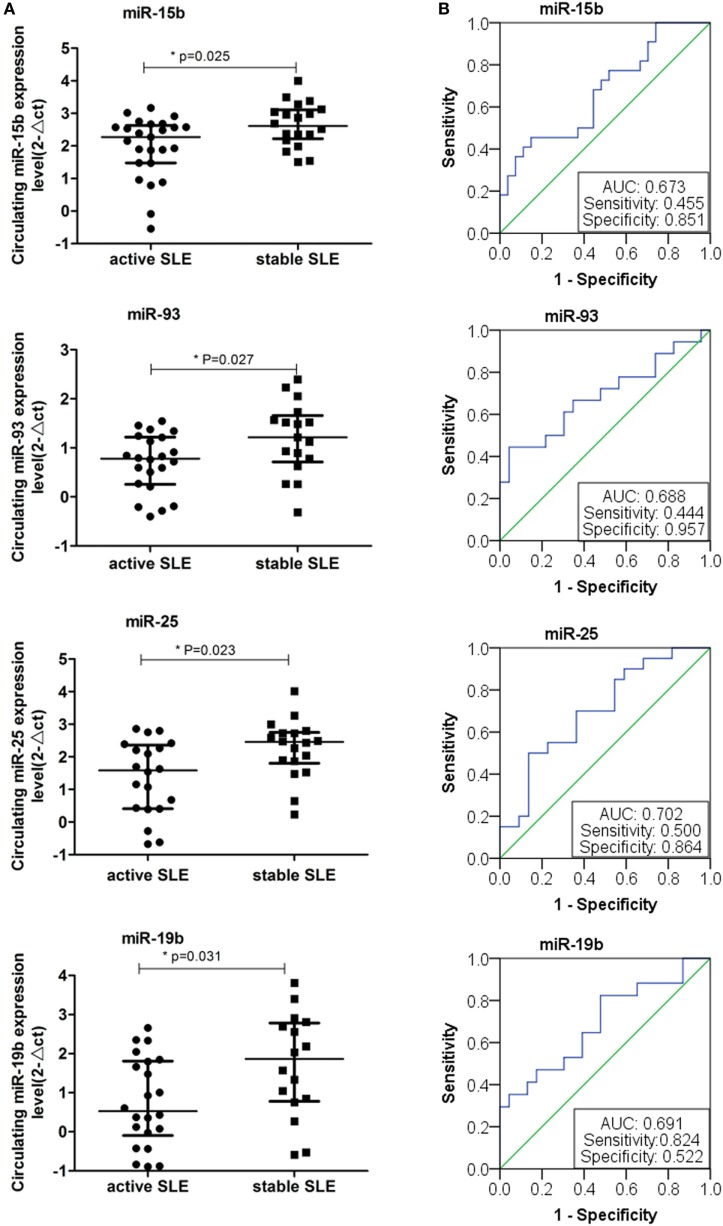
Expressions of four plasma microRNAs (miRNAs) in patients with active systemic lupus erythematosus (SLE). **(A)** Four plasma miRNAs expressed differentially in the active SLE and stable SLE patients. Expressions of selected miRNAs in the plasma obtained from patients with SLE (*n* = 50) were determined by RT-qPCR. The expression levels of miRNAs were normalized to cel-miR-39. **(B)** Receiver operating characteristic curve analysis of four plasma miRNAs expressed in the active SLE and stable SLE patients.

**Figure 6:**
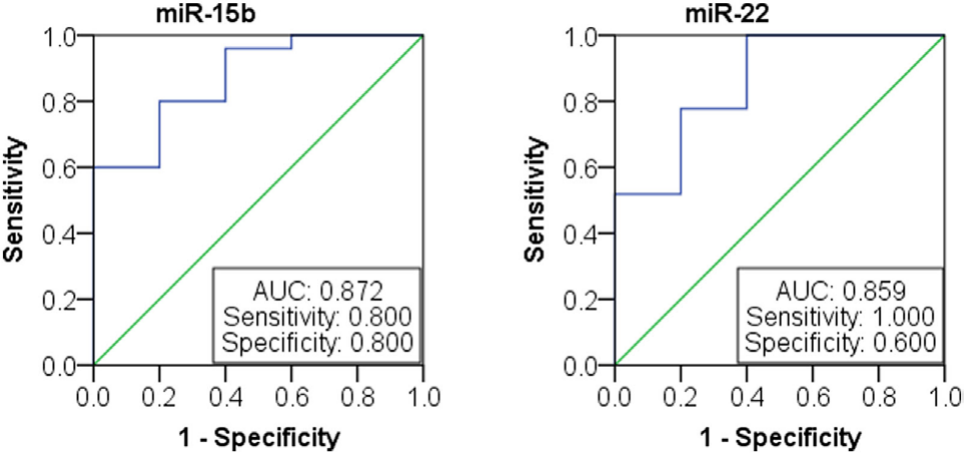
Receiver operating characteristic curve analysis of miR-15b and miR-22 microRNAs (miRNAs) expression in patients with lupus nephritis.

We next investigated the relationship between miRNAs expressions in plasma and established clinical variables of SLE patients (Table 6). There were only several miRNAs associated with some anti-nuclear antibodies although 14 selected miRNAs were SLE B cell-associated miRNAs. The expression levels of miR-19b, miR-22, miR-23a, and miR-25 in SLE patients with positive anti-β2GP1 were significantly higher than negative anti-β2GP1 patients (*P* < 0.05). The expression level of miR-15b was significantly higher in SLE patients with positive anti-SSB than those with negative anti-SSB (*P* = 0.014). There was no correlation between the expression of other miRNA and anti-U1RNP, anti-histone, anti-SSA, anti-Sm, and anti-Ribp in the plasma of SLE. Analysis of the relationship between expression of miRNAs in plasma and disease activity of SLE patients revealed that four miRNAs (miR-15b, miR-93, miR-25, and miR-19b) in the active group were significantly different from the stable group. Expressions of these four miRNAs in active state of SLE were all significantly lower than those in the stable SLE group (Figure 5A). According to the analysis of ROC curve (Figure 5B), AUCs for miR-15b, miR-93, miR-25, and miR-19b in determining active SLE patients were 0.619 (95% CI: 0.521–0.860), 0.702 (95% CI: 0.544–0.860), 0.688 (95% CI: 0.518–0.859), and 0.673 (95% CI: 0.521–0.825), respectively. In addition, data indicated that expressions of two plasma miRNAs (miR-15b and miR-22) were associated with renal injury of SLE. miR-15b and miR-22 were significantly lower in SLE patients with low eGFR (eGFR < 60 ml/min/1.73 m^2^) than those with better renal function (eGFR > 60 ml/min/1.73 m^2^) (*P* = 0.01 and 0.012, respectively). Analysis of ROC curve showed that the AUCs for miR-15b and miR-22 in determining SLE patients with renal damage (eGFR < 60 ml/min/1.73 m^2^) were 0.872 (95% CI: 0.714–1.000) and 0.859 (95% CI: 0.677–1.000), with sensitivity of 0.800 or 1.000 and specificity of 0.800 or 0.600, respectively (Figure 6). It did not have any significant association between expressions of plasma miRNAs and other SLE clinical variables (Table 7), except for miR-23a, miR-25, miR-16, miR-15b, miR-150, and miR-223. miR-23a was associated with gender, miR-25 was associated with CRP, and miR-15b, miR-150, and miR-223 were associated with ALT. Expression of plasma miR-16 was significantly lower in recurrent SLE patients than in SLE patients with first onset (*P* = 0.045). Analysis of treatment drugs showed that all SLE patients used steroids and 28 patients used immunosuppressive drugs among 50 SLE patients.

**Table 6:** Correlation of 14 microRNAs (miRNAs) with anti-nuclear antibodies in systemic lupus erythematosus patients.

miRNA	Anti-β2GP1		
	Negative (*n* = 8)	Positive (*n* = 16)	*P* value
miR-19b	58.85 (3.99, 163.99)	376.67 (108.53, 644.82)	0.049
miR-22	165.04 (19.78, 472.04)	345.58 (170.32, 520.86)	0.043
miR-23a	70.92 (17.95, 199.81)	141.87 (108.64, 175.11)	0.031
miR-25	136.57 (22.04, 436.89)	419.55 (308.56, 530.54)	0.016

**miRNA**	**Anti-SSB**		
	**Negative (***n*** = 20)**	**Positive (***n*** = 19)**	***P* value**

miR-15b	90.78 (29.75, 241.64)	369.38 (108.46, 653.18)	0.014

*The results are magnified 10,000 times*.

**Table 7:** Correlation of 14 microRNA (miRNA) expression with other clinical features of systemic lupus erythematosus.

miRNA	Sex				
	*N*	Male	*N*	Female	*P* value
miR-23a	8	49.45 (13.65, 146.85)	38	12.22 (2.72, 43.87)	0.046

**miRNA**	**CRP (mg/l)**				
	***N***	**<8**	***N***	**>8**	***P* value**

miR-25	18	101.34 (9.50, 559.01)	6	15.19 (0.23, 164.39)	0.046

**miRNA**	**ALT (U/l)**				
	***N***	**5–55**	***N***	**>55**	***P* value**

miR-15b	35	187.16 (34.70, 485.44)	3	479.41 (411.09, 5,287.22)	0.088
miR-150	32	20.54 (8.41, 64.96)	2	166.42 (94.92, 237.91)	0.048
miR-223	27	7.36 (1.73, 33.85)	2	1,258.80 (59.06, 2,458.75)	0.048

**miRNA**	**Incipient**				
	***N***	**Yes**	***N***	**No**	***P* value**

miR-16	3	28.14 (17.71, 265.16)	28	1.12 (0.28, 12.80)	0.045

### Double Validation of miR-15b as a Biomarker for SLE Patients With Disease Activity and LN

**Figure 7:**
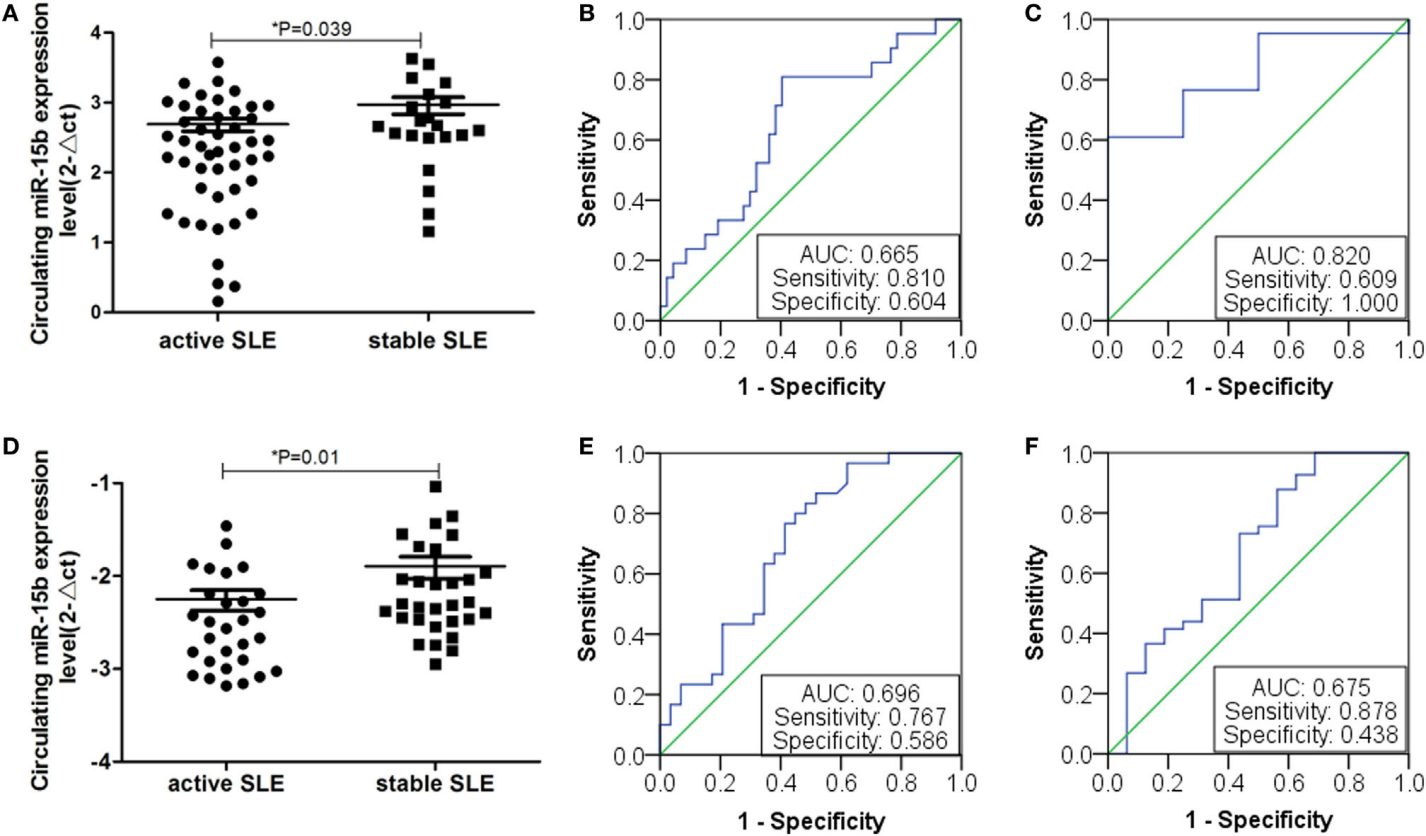
Expression of miR-15b in double validation group. **(A)** Plasma miR-15b expression of 69 samples from First Affiliated Hospital of Wenzhou Medical University. Plasma miR-15b was significantly different in active systemic lupus erythematosus (SLE) patients. **(B)** Receiver operating characteristic (ROC) curves with corresponding AUC for miR-15b in discriminating patients (*n* = 69) with active SLE from stable SLE. **(C)** Comparison of plasma miR-15b expression level in SLE patients (*n* = 69) with lupus nephritis (LN). **(D)** MiR-15b expressions of 80 serum samples from Ruijin Hospital of Shanghai Jiao Tong University. MiR-15b was significantly different in active SLE patients. **(E)** ROC curves with corresponding AUC for miR-15b in discriminating patients (*n* = 80) with active SLE from stable SLE. **(F)** Comparison of plasma miR-15b expression level in SLE patients (*n* = 69) with LN.

Based on the above analysis, miR-15b could act as a potential disease biomarker for SLE patients with disease activity and LN with low eGFR. Thus, to validate the results, we collected 69 SLE samples to further determine the expression of miR-15b. Results showed that miR-15b in the expression of SLE activity group was significant different from the stable group (*P* = 0.039, Figure 7A). According to the analysis of ROC curve (Figure 7B), miR-15b that separate active and stable state of the SLE patients with an AUC of the ROC curve of 0.665 (95% CI: 0.519–0.797), which determined the sensitivity (0.810) and specificity (0.604). Comparison of plasma miRNA expression level in patients with renal function revealed that the miR-15b in patients with low eGFR (eGFR < 60 ml/min/1.73 m^2^) was significantly decreased (*P* = 0.033); the analysis of ROC curve (Figure 7C) showed miR-15b to determine the AUC was 0.820 (95% CI: 0.662–0.978), which determined the sensitivity (0.609) and specificity (1.000). In addition, results obtained from another 80 SLE samples also showed that miR-15b in the expression of SLE activity group was significantly different from it in stable group (*P* = 0.01, Figure 7D). ROC curve analysis showed that AUC of miR-15b in predicting activity of the SLE patients was 0.696 (95% CI: 0.560–0.832), which determined the sensitivity at 0.767 and specificity at 0.586 (Figure 7E). Comparison plasma miRNA expression in patients with different eGFR level, miR-15b in patients with low eGFR (eGFR < 60 ml/min/1.73 m^2^) was significantly decreased (*P* = 0.028); based on the analysis of ROC curve, AUC of miR-15b in predicting LN with low GFR was 0.675 (95% CI: 0.508–0.843), which determined the sensitivity at 0.878 and specificity at 0.438 (Figure 7F). The results of treatment drugs showed that in the cohort of 69/80 SLE patients from Wenzhou/Shanghai, 67/77 patients used steroids and 46/61 patients used immunosuppressive drugs.

## Discussion

MicroRNA is very stable in blood circulation, and can be preserved for a long time without degradation *in vitro*, which makes miRNA very suitable for using as a new serum marker for a variety of diseases (11). The stability can be partially explained by the discovery of lipoprotein complexes, including small membrane vesicles of endocytic origin (called exosomes or microvesicles), high-density lipoprotein, and circulating Argonaute 2 complexes (23–25). Here, we used an ROC curve to evaluate the results and found that 14 miRNAs with clinical diagnostic value in the diagnosis of SLE are different from the HCs, six miRNAs with clinical diagnostic value are different between SLE and RA patients. The differential expression of miRNAs in plasma of SLE patients might be used as new biomarkers. Moreover, we analyzed the correlation between SLE-specific circulating miRNA and clinical features of patients with SLE, including autoantibodies and other related indicators. Results indicated that expressions of miR-19b, miR-25, miR-93, and miR-15b in SLE activity group were significantly lower than those in the SLE stable group. In addition, further analysis indicated that expressions of miR-15b and miR-22 correlated significantly with renal damage. Further diagnostic potential of miR-15b for SLE disease activity and LN was selected and validated on an independent validation set with 69 SLE patients and a cross-validation set with 80 SLE patients.

Circulating miRNA has been reported to be differentially expressed in SLE patients, but data are inconsistent in different studies, we reviewed them in PubMed and collected together in Table 8. Carlsen et al. screened 45 plasma miRNAs in two independent cohorts using quantitative RT-PCR assays (26). They found that seven miRNAs were statistically significant in plasma from SLE patients. Expression of miR-142-3p and miR-181a was increased while expression of miR-106a, miR-17, miR-20a, miR-203, and miR-92a was decreased. A 4-miRNA signature was diagnostic of SLE, and a specific subset of miRNA profiles was associated with nephritis. Wang et al. screened the plasma miRNA profiles of SLE patients by miRNA microarrays and identified eight miRNAs by RT-qPCR (27). They found that circulating levels of miR-16, miR-223, miR-23a, miR-15b, miR-150, and miR-25 were upregulated in patients with SLE in contrast to that expressions of circulating miR-155 and miR-92a were downregulated. Surprisingly, SLE-associated miR-155 was not significantly dysregulated in the plasma of both SLE and RA patients, compared with HCs. Using miRNA PCR array, Kim et al. found that nine miRNAs were differentially expressed between the SLE and HC (28). Three miRNAs (hsa-miR-30e-5p, hsa-miR-92a-3p, and hsa-miR-223-3p) were significantly upregulated in plasma of SLE patients (*P* = 0.048, *P* = 0.039, and *P* = 0.046, respectively). In our study, we found the plasma levels of 14 miRNAs were downregulated. The change of miR-92a in patients with SLE was consistent with previous studies (26, 27). We, together with Carlsen and colleagues, showed that expression of miR-20a in the SLE patients was downregulated. But expression of miR-155 in plasma was not significantly different between SLE patients and HCs.

**Table 8:** The comparison of circulating miRNAs in our study with other SLE studies.

miRNA	This study	Kim et al. (28)	Wang et al. (27)	Carlsen et al. (26)	Steen et al. (29)
Experiment subjects	Plasma	Plasma	Plasma	Plasma	Plasma
Number (SLE/HC)	199/20	70/40	30/20	131/143	29/40
miR-155	NS		↓	NS	
miR-150	↓	NS	NS	NS	↓
miR-20a	↓			↓	
miR-223	↓	↑	↑	NS	↑
miR-15b	↓		NS		
miR-16	↓		↑	NS	
miR-181a	↓			↑	
miR-23a	↓		NS		
miR-25	↓	NS	NS		
miR-92a	↓	↑	NS	↓	

*SLE/HC means SLE versus healthy control; number showed in the table means that we counted all the numbers of experiment subjects together*.

*NS, no significance; miRNAs, microRNAs; SLE, systemic lupus erythematosus; HC, healthy control*.

Using miRNAs sequencing, Navarro-Quiroz et al. comprehensively examine the plasma abundance of miRNAs in patients with class II, III, and IV lupus nephritis (LN II, LN III, and LN IV) compared with the plasma miRNA levels in lupus patients with no nephritis (LNN) or control healthy individuals (CTL) (30, 31). They found that expression of 89 miRNAs were significantly different in patients with LN, compared with CTL individuals. 17 miRNAs were differently abundant between patients with LN and LNN group. Compared with CTL individuals, the plasma level of miR-150 was upregulated in patients with LN III while miR-19b and miR-16 were downregulated in the patients with LN II. Comparison of LN II with LNN or LN IV with CTL, expression levels of miR-92a were all upregulated. In Wang and colleagues’ study of serum samples from HCs and early/late stage LN patients were used to analyze the expression of miRNAs by microarray. MiR-223 was upregulated and miR-22 was downregulated in the early LN patients versus HCs (32). On the other hand, miR-223, miR-19b, miR-22, miR-23a, miR-25, miR-92a, and miR-93 were all significantly downregulated in LN patients at the late stage compared to HCs (32).

Recently, several published studies have shown that miR-15b is involved in the pathologies of autoimmune disease. Liu et al. identified miR-15b as an important factor in Th17-associated effects and determined that expression of miR-15b was significantly downregulated in multiple sclerosis patients and in mice with experimental autoimmune encephalomyelitis (33). They also found that O-linked *N*-acetylglucosamine transferase is a potential target of miR-15b, enabling it to affect the transcriptional regulation of retinoic acid-related orphan receptor γT through O-linked *N*-acetylglucosamine glycosylation of NF-κB. Singh et al. reported that miR-15b/16 enhances the induction of regulatory T cells by regulating the expression of Rictor and mammalian target of rapamycin (34). Ren et al. used a B-cell profiling chip analysis and found that CyclinD3 was related to SLE and significantly elevated in SLE B cells (35). Furthermore, they demonstrated that activation of TLR7 dramatically increased CyclinD3 expression but significantly decreased miR-15b in B cells *in vitro*. They identified further that CyclinD3 is a direct target of miR-15b. Our results also showed that plasma miR-15b was decreased in patients with SLE, and was positively correlated with disease activity and LN with low eGFR. Thus, a larger sample size including different pathological types of LN, in a well-designed study will be promising for understanding of the functional role of miR-15b in future.

In conclusion, we have demonstrated here that differential expression of circulating miRNAs is a typical character between SLE patients and HCs. The plasma miR-15b correlated with SLE activity and LN may serve as a biomarker for SLE.

## Data Availability Statements

All relevant data are contained within the manuscript, all datasets for this study are included in the manuscript and the supplementary files. All the raw data of this manuscript are available by the authors, without undue reservation, to any qualified researcher.

## Ethics Statement

This study was carried out in accordance with the recommendations of the ethical standards of the responsible committee on human experimentation (the hospital of the First Affiliated Hospital of Wenzhou Medical University, and Ruijin Hospital of Shanghai Jiao Tong University School of Medicine, China). The protocol was approved by the hospital of the First Affiliated Hospital of Wenzhou Medical University, and Ruijin Hospital of Shanghai Jiao Tong University School of Medicine, China. All subjects gave written informed consent in accordance with the Declaration of Helsinki.

## Author Contributions

HZ, XH, and LY: performed the experiments, analyzed and interpreted the data, and drafted the manuscript. GG: performed the experiments and statistical analysis. XL, CC, LS, and BL: acquired the data and material support. XX and NC: made contribution to the conception and design, analyzed and interpreted the data, supervised the study, provided the project funding, revised the manuscript, and finally approved the version of the manuscript for publication.

## Conflict of Interest Statement

The authors declare that the research was conducted in the absence of any commercial or financial relationships that could be construed as a potential conflict of interest.

## References

[1] Frieri M. Mechanisms of disease for the clinician: systemic lupus erythematosus. Ann Allergy Asthma Immunol (2013) 110(4):228–32. 10.1016/j.anai.2012.12.010 23535084

[2] Aletaha D Neogi T Silman AJ Funovits J Felson DT Bingham CO III 2010 Rheumatoid arthritis classification criteria: an American College of Rheumatology/European League Against Rheumatism collaborative initiative. Arthritis Rheum (2010) 62(9):2569–81. 10.1002/art.27584 20872595

[3] Carthew RW Sontheimer EJ. Origins and mechanisms of miRNAs and siRNAs. Cell (2009) 136(4):642–55. 10.1016/j.cell.2009.01.035 19239886 PMC2675692

[4] Kim VN Han J Siomi MC Biogenesis of small RNAs in animals. Nat Rev Mol Cell Biol (2009) 10(2):126–39. 10.1038/nrm2632 19165215

[5] Pauley KM Cha S Chan EK. MicroRNA in autoimmunity and autoimmune diseases. J Autoimmun (2009) 32(3–4):189–94. 10.1016/j.jaut.2009.02.012 19303254 PMC2717629

[6] O’Connell RM Rao DS Chaudhuri AA Boldin MP Taganov KD Nicoll J Sustained expression of microRNA-155 in hematopoietic stem cells causes a myeloproliferative disorder. J Exp Med (2008) 205(3):585–94. 10.1084/jem.20072108 18299402 PMC2275382

[7] Taganov KD Boldin MP Chang KJ Baltimore D. NF-kappaB-dependent induction of microRNA miR-146, an inhibitor targeted to signaling proteins of innate immune responses. Proc Natl Acad Sci U S A (2006) 103(33):12481–6. 10.1073/pnas.0605298103 16885212 PMC1567904

[8] O’Connell RM Kahn D Gibson WS Round JL Scholz RL Chaudhuri AA MicroRNA-155 promotes autoimmune inflammation by enhancing inflammatory T cell development. Immunity (2010) 33(4):607–19. 10.1016/j.immuni.2010.09.009 20888269 PMC2966521

[9] Anolik JH. B cell biology: implications for treatment of systemic lupus erythematosus. Lupus (2013) 22(4):342–9. 10.1177/0961203312471576 23553777

[10] Miao CG Yang YY He X Huang C Huang Y Zhang L The emerging role of microRNAs in the pathogenesis of systemic lupus erythematosus. Cell Signal (2013) 25(9):1828–36. 10.1016/j.cellsig.2013.05.006 23707525

[11] Shen N Liang D Tang Y de Vries N Tak PP MicroRNAs – novel regulators of systemic lupus erythematosus pathogenesis. Nat Rev Rheumatol (2012) 8(12):701–9. 10.1038/nrrheum.2012.142 23070646

[12] Yan S Yim LY Lu L Lau CS Chan VS. MicroRNA regulation in systemic lupus erythematosus pathogenesis. Immune Netw (2014) 14(3):138–48. 10.4110/in.2014.14.3.138 24999310 PMC4079820

[13] Qu B Shen N. miRNAs in the pathogenesis of systemic lupus erythematosus. Int J Mol Sci (2015) 16(5):9557–72. 10.3390/ijms16059557 25927578 PMC4463604

[14] Willeit P Zampetaki A Dudek K Kaudewitz D King A Kirkby NS Circulating microRNAs as novel biomarkers for platelet activation. Circ Res (2013) 112(4):595–600. 10.1161/CIRCRESAHA.111.300539 23283721

[15] Mitchell PS Parkin RK Kroh EM Fritz BR Wyman SK Pogosova-Agadjanyan EL Circulating microRNAs as stable blood-based markers for cancer detection. Proc Natl Acad Sci U S A (2008) 105(30):10513–8. 10.1073/pnas.0804549105 18663219 PMC2492472

[16] Weber JA Baxter DH Zhang S Huang DY Huang KH Lee MJ The microRNA spectrum in 12 body fluids. Clin Chem (2010) 56(11):1733–41. 10.1373/clinchem.2010.147405 20847327 PMC4846276

[17] Murata K Yoshitomi H Tanida S Ishikawa M Nishitani K Ito H Plasma and synovial fluid microRNAs as potential biomarkers of rheumatoid arthritis and osteoarthritis. Arthritis Res Ther (2010) 12(3):R86. 10.1186/ar3013 20470394 PMC2911870

[18] Gregersen JW Jayne DR. B-cell depletion in the treatment of lupus nephritis. Nat Rev Nephrol (2012) 8(9):505–14. 10.1038/nrneph.2012.141 22801948

[19] Fang C Chaosheng C Huidi Z Xiangyang X Xiaokai D Li S Screening of the miRNAs related to disease activity in peripheral blood B lymphocytes of systemic lupus erythematosus patients. J Wenzhou Med Coll (2013) 2:106–11. 10.13771/j.cnki.33-1386/r.2013.02.011

[20] Hochberg MC Updating the American College of Rheumatology revised criteria for the classification of systemic lupus erythematosus. Arthritis Rheum (1997) 40(9):1725 10.1002/art.1780400928 9324032

[21] Arnett FC Edworthy SM Bloch DA McShane DJ Fries JF Cooper NS The American Rheumatism Association 1987 revised criteria for the classification of rheumatoid arthritis. Arthritis Rheum (1988) 31(3):315–24. 10.1002/art.1780310302 3358796

[22] Chen X Liang H Guan D Wang C Hu X Cui L A combination of Let-7d, Let-7g and Let-7i serves as a stable reference for normalization of serum microRNAs. PLoS One (2013) 8(11):e79652. 10.1371/journal.pone.0079652 24223986 PMC3818225

[23] Tabet F Vickers KC Cuesta Torres LF Wiese CB Shoucri BM Lambert G HDL-transferred microRNA-223 regulates ICAM-1 expression in endothelial cells. Nat Commun (2014) 5:3292. 10.1038/ncomms4292 24576947 PMC4189962

[24] Vickers KC Palmisano BT Shoucri BM Shamburek RD Remaley AT. MicroRNAs are transported in plasma and delivered to recipient cells by high-density lipoproteins. Nat Cell Biol (2011) 13(4):423–33. 10.1038/ncb2210 21423178 PMC3074610

[25] Cortez MA Bueso-Ramos C Ferdin J Lopez-Berestein G Sood AK Calin GA MicroRNAs in body fluids – the mix of hormones and biomarkers. Nat Rev Clin Oncol (2011) 8(8):467–77. 10.1038/nrclinonc.2011.76 21647195 PMC3423224

[26] Carlsen AL Schetter AJ Nielsen CT Lood C Knudsen S Voss A Circulating microRNA expression profiles associated with systemic lupus erythematosus. Arthritis Rheum (2013) 65(5):1324–34. 10.1002/art.37890 23401079 PMC6662589

[27] Wang H Peng W Ouyang X Li W Dai Y. Circulating microRNAs as candidate biomarkers in patients with systemic lupus erythematosus. Transl Res (2012) 160(3):198–206. 10.1016/j.trsl.2012.04.002 22683424

[28] Kim BS Jung JY Jeon JY Kim HA Suh CH. Circulating hsa-miR-30e-5p, hsa-miR-92a-3p, and hsa-miR-223-3p may be novel biomarkers in systemic lupus erythematosus. HLA (2016) 88(4):187–93. 10.1111/tan.12874 27596248

[29] Steen SO Iversen LV Carlsen AL Burton M Nielsen CT Jacobsen S The circulating cell-free microRNA profile in systemic sclerosis is distinct from both healthy controls and systemic lupus erythematosus. J Rheumatol (2015) 42(2):214–21. 10.3899/jrheum.140502 25399392

[30] Navarro-Quiroz E Pacheco-Lugo L Navarro-Quiroz R Lorenzi H Espana-Puccini P Diaz-Olmos Y Profiling analysis of circulating microRNA in peripheral blood of patients with class IV lupus nephritis. PLoS One (2017) 12(11):e0187973. 10.1371/journal.pone.0187973 29136041 PMC5685598

[31] Navarro-Quiroz E Pacheco-Lugo L Lorenzi H Diaz-Olmos Y Almendrales L Rico E High-throughput sequencing reveals circulating miRNAs as potential biomarkers of kidney damage in patients with systemic lupus erythematosus. PLoS One (2016) 11(11):e0166202. 10.1371/journal.pone.0166202 27835701 PMC5106044

[32] Wang W Mou S Wang L Zhang M Shao X Fang W Up-regulation of serum MiR-130b-3p level is associated with renal damage in early lupus nephritis. Sci Rep (2015) 5:12644. 10.1038/srep12644 26316103 PMC4551961

[33] Liu R Ma X Chen L Yang Y Zeng Y Gao J MicroRNA-15b suppresses Th17 differentiation and is associated with pathogenesis of multiple sclerosis by targeting O-GlcNAc transferase. J Immunol (2017) 198(7):2626–39. 10.4049/jimmunol.1601727 28228555

[34] Singh Y Garden OA Lang F Cobb BS. MicroRNA-15b/16 enhances the induction of regulatory T cells by regulating the expression of rictor and mTOR. J Immunol (2015) 195(12):5667–77. 10.4049/jimmunol.1401875 26538392 PMC4671309

[35] Ren D Liu F Dong G You M Ji J Huang Y Activation of TLR7 increases CCND3 expression via the downregulation of miR-15b in B cells of systemic lupus erythematosus. Cell Mol Immunol (2016) 13(6):764–75. 10.1038/cmi.2015.48 26144250 PMC5101438

