# Supplementary material for: B Cell-Related Circulating MicroRNAs With the Potential Value of Biomarkers in the Differential Diagnosis, and Distinguishment Between the Disease Activity and Lupus Nephritis for Systemic Lupus Erythematosus
Source: Front Immunol. 2018 Jun 29;9:1473. doi: 10.3389/fimmu.2018.01473 (PMC6033964; doi:10.3389/fimmu.2018.01473)
Supplement: Supplementary file 1 [file table_1.docx]

Table S1 The primers for miR-15b and cel-miR-39

| **Primer** | | **Sequence** |
| --- | --- | --- |
| miR-15b | RT stem-loop primer | GTCGTATCCAGTGCAGGGTCCGAGGTATTCGCACTGGATACGACTGTAAACC |
|  | Forward primer | AAGGTTCGTGGGTAGCAGCACATCAT |
| cel-miR-39 | RT stem-loop primer | GTCGTATCCAGTGCAGGGTCCGAGGTATTCGCACTGGATACGACCAAGCTGA |
|  | Forward primer | TTCGTGGGTCACCGGGTGTAAATC |
| Common reverse primer | | GCAGGGTCCGAGGTATTC |
