# Supplementary material for: B Cell-Related Circulating MicroRNAs With the Potential Value of Biomarkers in the Differential Diagnosis, and Distinguishment Between the Disease Activity and Lupus Nephritis for Systemic Lupus Erythematosus
Source: Front Immunol. 2018 Jun 29;9:1473. doi: 10.3389/fimmu.2018.01473 (PMC6033964; doi:10.3389/fimmu.2018.01473)
Supplement: Supplementary file 2 [file table_2.docx]

Table S2 miRNAs differentially involved in the development and function of the immune system.

| **miRNA** | **Function** | **Reference** | **Dyregulated in**  **immune disease** |
| --- | --- | --- | --- |
| miR-1224-3p | Associated with lupus nephritis | [[1](#_ENREF_1)] | SLE |
| miR-220b | Regulation of  human autoimmune regulator (AIRE) gene translation | [[2](#_ENREF_2)] | --- |
| miR-16 | Restriction of inflammatory  mediators production, regulator of  immunity through cooperation with  other miRNAs | [[3](#_ENREF_3)] | RA, IBD,  atherosclerosis,  osteoarthritis |
| miR-92a | Regulation of MMP-1 expression | [[4](#_ENREF_4)] | scleroderma  SLE |
| miR-155 | Regulation of bacterial and viral infection response in  macrophages/monocytes;  B and T cell  differentiation;  Regulator of germinal center B cell response;  Regulator of immunoglobulin  Production;  Regulator of acute inflammatory response | [[4](#_ENREF_4)] | SLE, RA, MS,  IBD,  atherosclerosis,  allergy, atopic  eczema |
| miR-20a | Involved in autoimmune  Phenomena; Regulates ASK1 expression and TLR4-dependent cytokine release in rheumatoid fibroblast-like synoviocytes; Binds to TF Mrna; Inhibit T cell activation genes | [[4](#_ENREF_4)] | autoimmune hemolytic anemiaRA,SLE,MS |
| miR-17 | Inhibit T cell activation genes Enhancer of T cell survival during development | [[4](#_ENREF_4)] | MS  --- |
| miR-223 | Maturation of promyelocytic  precursors into granulocytes  Negative regulator of the  proliferation/activation of  neutrophils | [[5](#_ENREF_5)] | RA |
| miR-494 | Targetd TNFSF14 | [[6](#_ENREF_6)] | MS |
| miR-126 | Enhancer of colony formation in vitro, may promote the production of downstream progenitors by HSCs | [[4](#_ENREF_4)] | IBD,  atherosclerosis,  allergy |
| miR-20b | Suppressing Th17 differentiation and targeting RORγt and STAT3 | [[7](#_ENREF_7)] | MS, experimental autoimmune encephalomyelitis |
| miR-25 | Modulate the TGF-β signaling pathway | [[8](#_ENREF_8)] | MS |
| miR-22 | Down-regulated the expression of PTEN;  Directly targeted the 3'-untranslated region of Cyr61 messenger RNA and inhibited Cyr61 expression | [[4](#_ENREF_4)] | SLE,RA |
| miR-27a | Linked to apoptosis and beta-cell networks | [[9](#_ENREF_9)] | --- |
| miR-15b | Significantly decreased | [[10](#_ENREF_10)] | MS |
| miR-23b | SuppressesIL-17-associatedautoimmune inflammation by targeting TAB2, TAB3 and IKK-α | [[11](#_ENREF_11)] | --- |
| miR-638 | Differentially expressed in lupus nephritis | [[1](#_ENREF_1)] | lupus nephritis |
| miR-146a | Modulates T-cell adhesion;A Dominant, Negative Regulator of the Innate Immune Response; Influencing the B-cell functions; Positive correlation of STAT1 | [[4](#_ENREF_4)] | SLE,RA  --- |
| miR-106b | Modulate the TGF-β signaling pathway | [[8](#_ENREF_8)] | MS |
| miR-19b | Represses the expression of Phosphatase and Tensin Homology (PTEN);  Contribute to increased TF expression | [[12](#_ENREF_12), [13](#_ENREF_13)] | MS,SLE |
| miR-29a | Contribute to cytokine-mediated β-cell dysfunction | [[14](#_ENREF_14)] | prediabetic |
| miR-26a | Regulates podocyte differentiation and cytoskeletal integrity | [[15](#_ENREF_15)] | Autoimmuneglomerulonephritis |
| miR-150 | B and T cell development | [[16](#_ENREF_16)] | RA,  atherosclerosis |
| miR-191 | Involved in vitiligo development | [[17](#_ENREF_17)] | autoimmune vitiligo |
| miR-24 | Potential diagnostic markers of RA | [[18](#_ENREF_18)] | RA |
| miR-181a | Targeting genes like Cxcr3 and Prkcd and Stat1 | [[19](#_ENREF_19)] | MS |
| miR-23a | Significantly decreased | [[10](#_ENREF_10)] | MS |
| miR-221 | Regulators of cell proliferation and engraftment | [[20](#_ENREF_20)] | psoriasis, atherosclerosis |
| miR-320a | Regulatd MMP-9 expression;Targeting mitogen-activated protein kinase 1 | [[21](#_ENREF_21), [22](#_ENREF_22)] | MS, myasthenia gravis |
| miR-342-3p | Significantly decreased in SLE patients with active nephritis | [[23](#_ENREF_23)] | SLE |
| miR-106a | Directly post-transcriptionally regulated  IL-10 | [[24](#_ENREF_24)] | --- |
| miR-93 | Underexpressed in  type 1 diabetes  patients | [[25](#_ENREF_25)] | --- |
| miR-140-3p | Downregulated in myasthenia gravis patients | [[26](#_ENREF_26)] | MG |

1. Te JL, Dozmorov IM, Guthridge JM, Nguyen KL, Cavett JW, Kelly JA et al. Identification of unique microRNA signature associated with lupus nephritis. PLoS One. 2010;5(5):0010344.

2. Matsuo T, Noguchi Y, Shindo M, Morita Y, Oda Y, Yoshida E et al. Regulation of human autoimmune regulator (AIRE) gene translation by miR-220b. Gene. 2013;530(1):19-25.

3. Lindsay MA. microRNAs and the immune response. Trends Immunol. 2008;29(7):343-51.

4. . !!! INVALID CITATION !!!

5. Johnnidis JB, Harris MH, Wheeler RT, Stehling-Sun S, Lam MH, Kirak O et al. Regulation of progenitor cell proliferation and granulocyte function by microRNA-223. Nature. 2008;451(7182):1125-9.

6. Jernas M, Malmestrom C, Axelsson M, Nookaew I, Wadenvik H, Lycke J et al. MicroRNA regulate immune pathways in T-cells in multiple sclerosis (MS). BMC Immunol. 2013;14(32):1471-2172.

7. Zhu E, Wang X, Zheng B, Wang Q, Hao J, Chen S et al. miR-20b suppresses Th17 differentiation and the pathogenesis of experimental autoimmune encephalomyelitis by targeting RORgammat and STAT3. J Immunol. 2014;192(12):5599-609.

8. De Santis G, Ferracin M, Biondani A, Caniatti L, Rosaria Tola M, Castellazzi M et al. Altered miRNA expression in T regulatory cells in course of multiple sclerosis. Journal of neuroimmunology. 2010;226(1-2):165-71. doi:10.1016/j.jneuroim.2010.06.009.

9. Nielsen LB, Wang C, Sorensen K, Bang-Berthelsen CH, Hansen L, Andersen ML et al. Circulating levels of microRNA from children with newly diagnosed type 1 diabetes and healthy controls: evidence that miR-25 associates to residual beta-cell function and glycaemic control during disease progression. Exp Diabetes Res. 2012;896362(10):5.

10. Fenoglio C, Ridolfi E, Cantoni C, De Riz M, Bonsi R, Serpente M et al. Decreased circulating miRNA levels in patients with primary progressive multiple sclerosis. Mult Scler. 2013;19(14):1938-42.

11. Zhu S, Pan W, Song X, Liu Y, Shao X, Tang Y et al. The microRNA miR-23b suppresses IL-17-associated autoimmune inflammation by targeting TAB2, TAB3 and IKK-alpha. Nat Med. 2012;18(7):1077-86.

12. Liu SQ, Jiang S, Li C, Zhang B, Li QJ. miR-17-92 cluster targets phosphatase and tensin homology and Ikaros Family Zinc Finger 4 to promote TH17-mediated inflammation. J Biol Chem. 2014;289(18):12446-56.

13. Teruel R, Perez-Sanchez C, Corral J, Herranz MT, Perez-Andreu V, Saiz E et al. Identification of miRNAs as potential modulators of tissue factor expression in patients with systemic lupus erythematosus and antiphospholipid syndrome. Journal of thrombosis and haemostasis : JTH. 2011;9(10):1985-92. doi:10.1111/j.1538-7836.2011.04451.x.

14. Roggli E, Gattesco S, Caille D, Briet C, Boitard C, Meda P et al. Changes in microRNA expression contribute to pancreatic beta-cell dysfunction in prediabetic NOD mice. Diabetes. 2012;61(7):1742-51.

15. Ichii O, Otsuka-Kanazawa S, Horino T, Kimura J, Nakamura T, Matsumoto M et al. Decreased miR-26a expression correlates with the progression of podocyte injury in autoimmune glomerulonephritis. PLoS One. 2014;9(10).

16. Zhou B, Wang S, Mayr C, Bartel DP, Lodish HF. miR-150, a microRNA expressed in mature B and T cells, blocks early B cell development when expressed prematurely. Proc Natl Acad Sci U S A. 2007;104(17):7080-5.

17. Shi YL, Weiland M, Lim HW, Mi QS, Zhou L. Serum miRNA expression profiles change in autoimmune vitiligo in mice. Exp Dermatol. 2014 Feb;23(2):140-2. doi: 10.1111/exd.12319.

18. Murata K, Furu M, Yoshitomi H, Ishikawa M, Shibuya H, Hashimoto M et al. Comprehensive microRNA analysis identifies miR-24 and miR-125a-5p as plasma biomarkers for rheumatoid arthritis. PLoS One. 2013;8(7).

19. Bergman P, James T, Kular L, Ruhrmann S, Kramarova T, Kvist A et al. Next-generation sequencing identifies microRNAs that associate with pathogenic autoimmune neuroinflammation in rats. J Immunol. 2013;190(8):4066-75.

20. Felli N, Fontana L, Pelosi E, Botta R, Bonci D, Facchiano F et al. MicroRNAs 221 and 222 inhibit normal erythropoiesis and erythroleukemic cell growth via kit receptor down-modulation. Proc Natl Acad Sci U S A. 2005;102(50):18081-6.

21. Aung LL, Mouradian MM, Dhib-Jalbut S, Balashov KE. MMP-9 expression is increased in B lymphocytes during multiple sclerosis exacerbation and is regulated by microRNA-320a. J Neuroimmunol. 2015;278:185-9.

22. Cheng Z, Qiu S, Jiang L, Zhang A, Bao W, Liu P et al. MiR-320a is downregulated in patients with myasthenia gravis and modulates inflammatory cytokines production by targeting mitogen-activated protein kinase 1. J Clin Immunol. 2013;33(3):567-76.

23. Carlsen AL, Schetter AJ, Nielsen CT, Lood C, Knudsen S, Voss A et al. Circulating microRNA expression profiles associated with systemic lupus erythematosus. Arthritis Rheum. 2013;65(5):1324-34.

24. Quinn SR, O'Neill LA. The role of microRNAs in the control and mechanism of action of IL-10. Curr Top Microbiol Immunol. 2014;380:145-55.

25. Salas-Perez F, Codner E, Valencia E, Pizarro C, Carrasco E, Perez-Bravo F. MicroRNAs miR-21a and miR-93 are down regulated in peripheral blood mononuclear cells (PBMCs) from patients with type 1 diabetes. Immunobiology. 2013;218(5):733-7.

26. Nogales-Gadea G, Ramos-Fransi A, Suarez-Calvet X, Navas M, Rojas-Garcia R, Mosquera JL et al. Analysis of serum miRNA profiles of myasthenia gravis patients. PLoS One. 2014;9(3).
