# Supplementary material for: B Cell-Related Circulating MicroRNAs With the Potential Value of Biomarkers in the Differential Diagnosis, and Distinguishment Between the Disease Activity and Lupus Nephritis for Systemic Lupus Erythematosus
Source: Front Immunol. 2018 Jun 29;9:1473. doi: 10.3389/fimmu.2018.01473 (PMC6033964; doi:10.3389/fimmu.2018.01473)
Supplement: Supplementary file 3 [file table_3.docx]

Table S3 Gene in Chip of circulating miRNA

| hsa-miR-107 | hsa-miR-150 | hsa-miR-1915 | hsa-miR-24 | hsa-miR-92a |
| --- | --- | --- | --- | --- |
| has-miR-320a | hsa-miR-155 | hsa-miR-19b | hsa-miR-25 | hsa-miR-93 |
| hsa-miR-103 | hsa-miR-15b | hsa-miR-20a | hsa-miR-26a | hsa-let-7d |
| hsa-miR-106a | hsa-miR-16 | hsa-miR-20b | hsa-miR-27a | Hsa-let-7j |
| hsa-miR-106b | hsa-miR-17 | hsa-miR-22 | hsa-miR-29a | Hsa-let-7i |
| hsa-miR-1224-3p | hsa-miR-181a | hsa-miR-220b | hsa-miR-342-3p | U6 |
| hsa-miR-1249 | hsa-miR-181b | hsa-miR-221 | hsa-miR-378 | Cel-miR-39 |
| hsa-miR-126 | hsa-miR-185 | hsa-miR-223 | hsa-miR-494-3p | GDC |
| hsa-miR-140-3p | hsa-miR-187 | hsa-miR-23a | hsa-miR-638 |  |
| hsa-miR-146a | hsa-miR-191 | hsa-miR-23b | hsa-miR-652 |  |
