# Supplementary material for: B Cell-Related Circulating MicroRNAs With the Potential Value of Biomarkers in the Differential Diagnosis, and Distinguishment Between the Disease Activity and Lupus Nephritis for Systemic Lupus Erythematosus
Source: Front Immunol. 2018 Jun 29;9:1473. doi: 10.3389/fimmu.2018.01473 (PMC6033964; doi:10.3389/fimmu.2018.01473)
Supplement: Supplementary file 4 [file table_4.docx]

Table S4 42 miRNA accession and sequence

| **microRNAs** | **Accession** | **Mature microRNA sequence** |
| --- | --- | --- |
| hsa-miR-1224-3p | MIMAT0005459 | CCCCACCUCCUCUCUCCUCAG |
| hsa-miR-1249 | MIMAT0005901 | ACGCCCUUCCCCCCCUUCUUCA |
| hsa-miR-1915 | MIMAT0007892 | CCCCAGGGCGACGCGGCGGG |
| hsa-miR-220b | MIMAT0000318 | UAAUACUGCCUGGUAAUGAUGA |
| hsa-miR-223 | MIMAT0000280 | UGUCAGUUUGUCAAAUACCCCA |
| hsa-miR-494-3p | MIMAT0002816 | UGAAACAUACACGGGAAACCUC |
| hsa-miR-126 | MIMAT0000445 | UCGUACCGUGAGUAAUAAUGCG |
| hsa-miR-20b | MIMAT0001413 | CAAAGUGCUCAUAGUGCAGGUAG |
| hsa-miR-25 | MIMAT0000081 | CAUUGCACUUGUCUCGGUCUGA |
| hsa-miR-22 | MIMAT0000077 | AAGCUGCCAGUUGAAGAACUGU |
| hsa-miR-652 | MIMAT0003322 | AAUGGCGCCACUAGGGUUGUG |
| hsa-miR-378 | MIMAT0000731 | CUCCUGACUCCAGGUCCUGUGU |
| hsa-miR-181b | MIMAT0000257 | AACAUUCAUUGCUGUCGGUGGGU |
| hsa-miR-27a | MIMAT0000084 | UUCACAGUGGCUAAGUUCCGC |
| hsa-miR-15b | MIMAT0000417 | UAGCAGCACAUCAUGGUUUACA |
| hsa-miR-23b | MIMAT0000418 | AUCACAUUGCCAGGGAUUACC |
| hsa-miR-638 | MIMAT0003308 | AGGGAUCGCGGGCGGGUGGCGGCCU |
| hsa-miR-146a | MIMAT0000449 | UGAGAACUGAAUUCCAUGGGUU |
| hsa-miR-106b | MIMAT0000680 | UAAAGUGCUGACAGUGCAGAU |
| hsa-miR-19b | MIMAT0000074 | UGUGCAAAUCCAUGCAAAACUGA |
| hsa-miR-185 | MIMAT0000455 | UGGAGAGAAAGGCAGUUCCUGA |
| hsa-miR-29a | MIMAT0000086 | UAGCACCAUCUGAAAUCGGUUA |
| hsa-miR-26a | MIMAT0000082 | UUCAAGUAAUCCAGGAUAGGCU |
| hsa-miR-150 | MIMAT0000451 | UCUCCCAACCCUUGUACCAGUG |
| hsa-miR-103 | MIMAT0000101 | AGCAGCAUUGUACAGGGCUAUGA |
| hsa-miR-92a | MIMAT0000092 | UAUUGCACUUGUCCCGGCCUGU |
| hsa-miR-20a | MIMAT0000075 | UAAAGUGCUUAUAGUGCAGGUAG |
| hsa-miR-16 | MIMAT0000069 | UAGCAGCACGUAAAUAUUGGCG |
| hsa-miR-191 | MIMAT0000440 | CAACGGAAUCCCAAAAGCAGCUG |
| hsa-miR-107 | MIMAT0000104 | AGCAGCAUUGUACAGGGCUAUCA |
| hsa-miR-24 | MIMAT0000080 | UGGCUCAGUUCAGCAGGAACAG |
| hsa-miR-181a | MIMAT0000256 | AACAUUCAACGCUGUCGGUGAGU |
| hsa-miR-23a | MIMAT0000078 | AUCACAUUGCCAGGGAUUUCC |
| hsa-miR-155 | MIMAT0000646 | UUAAUGCUAAUCGUGAUAGGGGU |
| hsa-miR-17 | MIMAT0000070 | CAAAGUGCUUACAGUGCAGGUAG |
| hsa-miR-221 | MIMAT0000278 | AGCUACAUUGUCUGCUGGGUUUC |
| has-miR-320a | MIMAT0000510 | AAAAGCUGGGUUGAGAGGGCGA |
| hsa-miR-342-3p | MIMAT0000753 | UCUCACACAGAAAUCGCACCCGU |
| hsa-miR-106a | MIMAT0000103 | AAAAGUGCUUACAGUGCAGGUAG |
| hsa-miR-93 | MIMAT0000093 | CAAAGUGCUGUUCGUGCAGGUAG |
| hsa-miR-140-3p | MIMAT0004597 | UACCACAGGGUAGAACCACGG |
| hsa-miR-1224-4p | MIMAT0006343 | CCCCACCUCCUCUCUCCUCAG |
