# Supplementary material for: B Cell-Related Circulating MicroRNAs With the Potential Value of Biomarkers in the Differential Diagnosis, and Distinguishment Between the Disease Activity and Lupus Nephritis for Systemic Lupus Erythematosus
Source: Front Immunol. 2018 Jun 29;9:1473. doi: 10.3389/fimmu.2018.01473 (PMC6033964; doi:10.3389/fimmu.2018.01473)
Supplement: Supplementary file 5 [file table_5.docx]

Table S5. The comparison of miRNAs in our study with other lupus nephritis studies

| miRNA | This study | Navarro-Quiroz E[[1](#_ENREF_1)] | Wang W[[2](#_ENREF_2)] | Dai[[3](#_ENREF_3)] | Carlsen AL [[4](#_ENREF_4)] | Zhou H[[5](#_ENREF_5)] |
| --- | --- | --- | --- | --- | --- | --- |
| Experiment subjects | plasma | plasma | serum | Kidney biopsies | plasma | kidney biopsies |
| Number | 199/20  SLE/HC | 20/40/40/40/40  LN Ⅱ/Ⅲ/IV/LNN/CTL | 60/36  LN/HC | 5/3  LNⅡ/NC | 21/11  AN/SN | 8  LN patients |
| miR-150 |  | ↑ (LNⅢ vs CTL) |  | ↓(LN vs NC) |  | ↑  （high vs low CI） |
| miR-20a |  |  |  |  | ↓(AN) |  |
| miR-223 |  |  | ↑ (LNE vs CTL) and  ↓ (LNL vs CTL) | ↓(LN vs NC) | ↓(AN) |  |
| miR-15b | ↓ |  |  | ↑(LN vs NC) |  |  |
| miR-16 |  | ↓ (LNⅡ vs CTL) |  |  |  |  |
| miR-19b |  | ↓ (LNⅡ vs CTL) | ↓ (LNL vs CTL) |  |  |  |
| miR-22 | ↓ |  | ↓ (LNE vs CTL) and  (LNL vs CTL) |  |  |  |
| miR-23a |  |  | ↓ (LNL vs CTL) |  |  |  |
| miR-25 |  |  | ↓ (LNL vs CTL) |  |  |  |
| miR-92a |  | ↑ (LNⅣ vs CTL) and (LNⅡ vs LNN) | ↓ (LNL vs CTL) |  |  |  |
| miR-93 |  |  | ↓ (LNL vs CTL) |  |  |  |

*AN: active nephritis; SN: stable nephritis; HC/NC: healthy/ normal control; LN-IV/Ⅲ/Ⅱ: Class IV/Ⅲ/Ⅱ; lupus nephritis; LNN: patients with lupus without nephritis ; CTL: healthy individuals; CI: chronicity index

1. Navarro-Quiroz E, Pacheco-Lugo L, Lorenzi H, Diaz-Olmos Y, Almendrales L, Rico E et al. High-Throughput Sequencing Reveals Circulating miRNAs as Potential Biomarkers of Kidney Damage in Patients with Systemic Lupus Erythematosus. PloS one. 2016;11(11):e0166202. doi:10.1371/journal.pone.0166202.

2. Wang W, Mou S, Wang L, Zhang M, Shao X, Fang W et al. Up-regulation of Serum MiR-130b-3p Level is Associated with Renal Damage in Early Lupus Nephritis. Sci Rep. 2015;5:12644. doi:10.1038/srep12644.

3. Dai Y, Sui W, Lan H, Yan Q, Huang H, Huang Y. Comprehensive analysis of microRNA expression patterns in renal biopsies of lupus nephritis patients. Rheumatology international. 2009;29(7):749-54. doi:10.1007/s00296-008-0758-6.

4. Carlsen AL, Schetter AJ, Nielsen CT, Lood C, Knudsen S, Voss A et al. Circulating microRNA expression profiles associated with systemic lupus erythematosus. Arthritis and rheumatism. 2013;65(5):1324-34. doi:10.1002/art.37890.

5. Zhou H, Hasni SA, Perez P, Tandon M, Jang SI, Zheng C et al. miR-150 promotes renal fibrosis in lupus nephritis by downregulating SOCS1. Journal of the American Society of Nephrology : JASN. 2013;24(7):1073-87. doi:10.1681/asn.2012080849.
